# Outcome of eight working dogs with fibrotic myopathy following extracorporeal shockwave and rehabilitation therapy: a case series

**DOI:** 10.3389/fvets.2023.1258319

**Published:** 2024-01-08

**Authors:** Frank C. Tsai, Leilani X. Alvarez

**Affiliations:** Department of Integrative and Rehabilitative Medicine, Schwarzman Animal Medical Center, New York, NY, United States

**Keywords:** fibrotic myopathy, extracorporeal shockwave therapy, gracilis, semitendinosus, German shepherd, working dogs, muscle contracture

## Abstract

**Introduction:**

Fibrotic myopathy of the gracilis, semitendinosus, and semimembranosus is described primarily in working German Shepherd dogs. The purpose of this case series is to describe the rehabilitation modalities and treatments utilized in working dogs with fibrotic myopathy and the time frame they were able to continue working.

**Methods:**

Medical records of patients with hindlimb lameness that were presented to the Schwarzman Animal Medical Center in New York City from 2012 to 2023 were retrospectively searched. Signalment, history, clinical evaluation, gait analysis, goniometry of stifles, and follow-up evaluation were compared among patients. Ten male working dogs met inclusion criteria. Extracorporeal Shockwave Therapy (ECSWT) was administered under sedation or general anesthesia. Rehabilitation therapy (RT), including massage, hamstring stretch, photobiomodulation, pulsed electromagnetic field therapy, warm compress, therapeutic ultrasound, underwater treadmill, and therapeutic exercises, were performed once every one to 3 weeks with varying protocols according to patient assessments. Follow-up phone calls and emails were conducted to determine long-term outcome.

**Results:**

On average, dogs were able to work full-time for 32.1 months (range 6–82; SD 23.6) from the time of diagnosis. No activity limitation was reported by the owners/handlers.

**Discussion:**

This report is the first to describe non-invasive medical treatments that may extend the working ability of dogs diagnosed with fibrotic myopathy. Further prospective randomized controlled studies are needed to demonstrate the efficacy of ECSWT and RT for treating fibrotic myopathy.

**Conclusion:**

The results of this retrospective study suggest that the combination of ECSWT and RT may allow working dogs with fibrotic myopathy to continue their working capacity for an extended period of time.

## Introduction

Fibrotic myopathy, specifically of the gracilis, semitendinosus, or semimembranosus, is an uncommon disease in dogs. It has been reported in the German shepherd, Belgian Malinois, Doberman Pinscher, Rottweiler, and Old English Sheepdog (1–6). Predominantly young adult, male German shepherd working dogs, or dogs with active lifestyles, are reported (1, 5). The exact etiology is still unknown but various causes are proposed. Muscle trauma, either from repeated muscle strains or a single event is likely the primary cause (1, 2, 4). Other explanations include compartment syndrome, fractures, infection (Neospora), immune-mediated, neuropathy, and immobilization (7). Regardless of the original insult, disease progression is usually associated with breakdown of muscle fibers and replacement by fibrous tissue and subsequent muscle contracture and loss of elasticity (5, 6).

Typically, acute trauma/injury is not reported by the owners/handlers in most cases (4, 5). Gait abnormality is usually the only symptom reported with no associated pain and can sometimes be mistaken as neurological disease. Except for acute muscle strain injury, improvement usually is not observed with pain medications (including non-steroidal anti-inflammatory drugs, opioids, etc.), or rest (4, 8).

Although trauma, whether a single event or repeated microtrauma, has been suspected to be the cause of fibrotic myopathy of gracilis/semitendinosus in dogs, etiopathogenesis remains elusive. Adding to the complexity, in racing Greyhounds, rupture of the gracilis muscle results in some degree of contracture and adhesion, but the gait is not affected (1), suggesting a genetic predisposition of German Shepherds and Malinois breeds. Fibrotic myopathy of the gracilis, semimembranosus, and/or semitendinosus muscles causes a distinctive gait pattern—with a shortened stride, rapid elastic medial rotation of the paw, external rotation of the hock, and internal rotation of the stifle during mid- to late-swing phase of the stride (3–5, 9). The lameness might be more noticeable at a trot. On physical examination, a taut band can be palpated, depending on which muscle is affected. Pain may or may not be noticed on muscle palpation. Joint range of motion is expected to be decreased during hip abduction, stifle extension, and tarsal extension (4, 9).

Medical and surgical management has been described with guarded prognosis (3–5). Medical management, including therapeutic ultrasound, immunosuppressive dose of corticosteroids, D-Penicillamine and colchicine did not improve the condition (3, 4). Post-surgery rehabilitation therapy, including cross-fiber friction massage, passive joint range of motion, and controlled exercises did not yield sustained improvement either (3, 10). Previous reports of surgical interventions resulted in immediate improvement, if not full resolution of the lameness, but the lameness was expected to recur in 2–4 months (1, 4, 5).

In one study (8), authors demonstrated that adipose-derived mesenchymal stem cells could improve or help prevent progression of fibrosis and muscle contracture in dogs with semitendinosus myopathy. It is worth noting the cases were treated early and only 3/10 cases had evidence of scar (fibrous) tissue formation (true fibrotic myopathy).

Extracorporeal Shockwave Therapy (ECSWT) has been used to treat various musculoskeletal conditions, including bone healing, tendinopathy, patellar desmopathy, acute/subacute myopathies, lumbosacral disease/pain, and osteoarthritis (11). It has been documented that ECSWT could noninvasively, effectively, and safely prevent the formation of arthrofibrosis during knee repair in rabbits (12).

In one human study, ECSWT was comparable to intralesional steroid injection for treatment of keloid scars (13). A systematic review for the safety and efficacy of treating post-burn scars suggests that the combination of ECSWT and comprehensive rehabilitation therapy (RT) had better therapeutic effect on post-burn pathological scars than RT alone, without obvious side effects (14). ECSWT has been used with variable success to treat fibromatosis diseases in people, including plantar (Ledderhose disease), palmar (Dupuytren’s disease), and penile fibromatosis (Peyronie’s disease) (15–18).

Although dogs can remain active in spite of the pathognomonic gait/lameness (1), the muscle contracture from fibrosis can be career-ending for working police dogs (8). To date, the effect of ECSWT on fibrotic myopathy in working dogs, and specifically, the ability to continue to work, has not been described.

The aim of this case series was to document the treatment and outcomes of ECSWT and RT on working dogs with hindlimb fibrotic myopathy, and to report the length of time they were able to continue working full-time after diagnosis.

Our hypothesis was that ECSWT and RT would enable working dogs with hindlimb fibrotic myopathy to continue working full-time after the time of diagnosis for an average of 1 year (12 months) or longer.

## Materials and methods

Medical records of canine patients with a chief complaint of hind limb lameness presented to the Schwarzman Animal Medical Center (AMC) in New York City between January 2012 to June 2023 were retrospectively searched in the electronic medical record database and evaluated. The terms, “fibrotic myopathy” and “canine” were searched. Exclusion criteria included pet dogs (non-working), forelimb fibrotic myopathy, or a diagnosis that was not conclusive or after retirement. Signalment, history, clinical evaluation, thigh girth measurement, gait analysis (both subjective and objective if available), goniometry of stifles, and follow-up evaluation were compared. All ECSWT was performed with the same electrohydraulic machine (VersaTron, PulseVet, Alpharetta, GA, United States). The setting for the ECSWT was: 1,000 pulses at energy level E6 (0.15 mJ/mm^2^; however, energy densities are not comparable across different ECSWT devices) (11) to each affected muscle using 20 mm trode. Treatments were administered under sedation every 2 weeks for a total of 1–3 treatments.

Thigh girth measurements were performed using a Gulick II tape measure (Country Technology, Inc. Gays Mills, WI, United States). It was measured in a consistent manner over the greater trochanter in standing posture. Subjective lameness was graded as none (I), mild (II), moderate (III), or severe (IV) (19). Objective gait analysis was conducted using a pressure-sensitive walkway system (Gait4Dog, CIR Systems, Franklin, NJ, United States) with a minimum of three consistent gait passes.

All patients had either musculoskeletal ultrasound (MSK US) examination performed by a board-certified radiologist, magnetic resonance imaging (MRI; reviewed by a board-certified radiologist), or clinical evaluation by a board-certified specialist in either sport medicine and rehabilitation, surgery, or neurology. The criteria for diagnosis of gracilis/semitendinosus/semimembranosus fibrotic myopathy included firm taut bands on palpation of the caudomedial thigh, identifying the origin/insertion of the fibrotic muscles, and the pathognomonic gait (as described previously). Additionally, the degree of hamstrings flexibility was noted.

The patients were sedated prior to the administration of ECSWT, except two patients (one was performed under general anesthesia; Midazolam 0.5 mg/kg IV, Propofol 3 mg/kg IV, Isoflurane and the other was performed non-sedated), under a protocol chosen by the supervising clinician (Nalbuphine 0.2 mg/kg IV/IM + Midazolam 0.2 mg/kg IV/IM+ Propofol 2.4–3.5 mg/kg IV; Nalbuphine 0.2 mg/kg IV/IM+ Dexmedetomidine 7–10 mcg/kg IV/IM; Dexmedetomidine 5–7 mcg/kg IV/IM + Hydromorphone 0.1 mg/kg IV or Methadone 0.1 mg/kg IV + Dexmedetomidine 6 mcg/kg IV).

Rehabilitation therapy included manual therapies (massage, passive joint range of motion of the hip, stifle joints, stretching of the gracilis/semimembranosus/semitendinosus), photobiomodulation to hamstrings (Machine 1: Pain and Trauma setting, 10 W, 2,600–3,120 joules total, ~7–9 J/cm^2^, Companion, LiteCure LLC, Newark, DE, United States; Machine 2: Wound healing setting, 101–151 joules total, 2 J/cm^2^, MLS, Mphi VET, ASALASER, Arcugnano, Italy), Pulsed Electromagnetic Field (PEMF) therapy (15 min over hamstrings; Assisi loop, Assisi Animal Health, Santa Fe, NM, United States), warm compress (10–15 min over hamstrings before massage and stretching), customized therapeutic exercises and underwater treadmill walking, individualized according to patient assessment. Typically, RT lasted about 50–60 min and was performed once weekly.

Therapeutic ultrasound (Chattanooga, DJO LLC, Vista, CA, United States) was used over affected muscles (5 cm^2^ head, 1.0 MHz, 50–100%, 0.5–1 W/cm^2^ for 7–10 min) in some patients after the course of ECSWT. Duty cycle was chosen for non-thermal (50%) and thermal effects (100%).

Each patient had follow-up evaluations with the rehabilitation therapists/specialists or primary care veterinarians. Outcome measurements were performed by either a Diplomate of the American College of Veterinary Sports Medicine and Rehabilitation (DACVSMR), a certified rehabilitation veterinarian or technician, or internship-trained primary care veterinarian at the AMC. Follow-up phone calls/emails were conducted by the primary author. Patients were followed-up either by in-person examination or via phone call to report the status of working ability.

## Results

A total of 17 cases were identified in the medical record system who had a diagnosis of gracilis/semimembranosus/semitendinosus fibrotic myopathy. Five non-working dogs were excluded. Two additional working dogs were excluded because the diagnosis of fibrotic myopathy was after retirement. Out of the 10 cases included in this case series, there were nine police dogs, and one guide dog.

All cases evaluated were male German shepherd dogs, except one male Belgian Malinois with ages ranging from 2.5 to 9.8 years old at the time of diagnosis (mean 5.5 SD 1.8) (Table 1). Six were castrated and 4 were intact (Table 1). The ages of 8 patients who received ECSWT and RT (treatment group) ranged from 2.5 to 6.3 years old (mean 5.0 SD 1.2) (Table 1). Two patients who did not receive ECSWT or RT were 5 and 9.8 years old at the time of diagnosis (mean 7.4 SD 2.4) (Table 1).

**Table 1:** Patient demographics.

Patient	1	2	3	4	5	6	7	8	Mean SD	9	10	Mean SD	Total mean SD
Age (years) at the time of diagnosis	6	6	5.5	2.5	6.25	5.4	3.8	4.75	5.0 1.2	5	9.83	7.4 2.4	5.5 1.8
Breed	GSD	GSD	GSD	GSD	GSD	GSD	GSD	Mal		Shep	GSD		
Neuter status	N	N	N	N	I	I	N	N		I	I		
Job description	PD	PD	PD	GD	PD	PD	PD	PD		PD	PD		
Muscles affected	G, SM, B	G, B	G, B	G, SM, L	G, SM, L	G or SM, B	SM, B	G, B		G, SM, B	ST, B		

All were male dogs. GSD, German shepherd dog; Mal, Belgian Malinois; Shep, Shepherd—not specified; N, Neutered; I, Intact; PD, Police Dog; GD, Guide Dog for the Blinds; G, Gracilis Muscle; SM, Semimembranosus Muscle; ST, Semitendinosus Muscle; B, Bilateral; R, Right; L, Left.

No specific activities or events were identified by the owners/handlers that led to the unusual gait.

Three of the 8 patients in the treatment group had MRI to confirm the diagnosis of fibrotic myopathy of gracilis or semimembranosus. One patient had MSK US to confirm the diagnosis of fibrotic myopathy. The rest of the patients were diagnosed based on palpation of fibrotic bands and pathognomonic gait. One of the two patients who did not receive ECSWT or RT (non-treatment group) was diagnosed with fibrotic myopathy before his retirement per the handler 2 years prior to presentation. No referral record could be obtained (the previous clinic was closed and bought by another practice that did not keep his complete medical record) to verify the diagnosis. The diagnosis of fibrotic myopathy was confirmed by a board-certified surgeon at the AMC (2 years after his retirement and 4 years after diagnosis, through palpation and pathognomonic gait).

All 8 patients of the treatment group received ECSWT. Six of the patients had the recommended 3 treatments spaced 2 weeks apart and 2 had just one treatment. All 8 patients also received customized therapeutic exercises and manual therapies (Table 2). Other therapeutic rehabilitation therapies administered to the dogs are outlined in Table 2.

**Table 2:** Rehabilitation treatments performed in dogs with fibrotic myopathy.

Patient treatment	1	2	3	4	5	6	7	8	9	10
ECSWT (# of treatments)	+ (3)	+ (3)	+ (3)	+ (1)	+ (3)	+ (1)	+ (3)	+ (3)	0	0
Muscles treated by ECSWT	G, SM, B	G, B	G, B	G, SM, L	G, SM, B	G-B, SM- R	G, SM, B	G, L	−	−
Manual therapy (passive joint range of motion, hamstring stretching, massage)	+	+	+	+	+	+	+	+	None	None
Photobiomodulation	+	+	+	+	+	−	−	−	−	−
Superficial heat	+	+	−	−	−	−	+	−	−	−
Therapeutic ultrasound	+	+	−	+	+	+	+	+	−	−
PEMF	−	−	−	−	+	+	−	−	−	−
Underwater treadmill	+	+	+	+	−	+	+	+	−	−
Land treadmill	−	−	−	−	+	−	−	−	−	−
Therapeutic exercises	+	+	+	+	+	+	+	+	−	−
RT Frequency (every_weeks)	1–3	1–3	1–2	1–2	1–2	1	1	1	−	−
RT total # of treatments	10	77	4	392	18	3	9	18	−	−

+, performed; −, not performed; ECSWT, Extracorporeal Shockwave Therapy; PEMF, Pulsed Electromagnetic Field; RT, Rehabilitation Therapy; G, Gracilis Muscle; SM, Semimembranosus Muscle; ST, Semitendinosus Muscle; B, Bilateral; R, Right; L, Left.

For the 2 patients in the non-treatment group, no objective outcomes were available because they did not return for follow-up with reference to the fibrotic myopathy diagnosis or treatment.

Regarding the treatment outcomes, 2 out of the 8 patients were noted to have a softer muscle belly of the affected muscles after treatment with ECSWT. Others did not have noticeable change.

Stifle range of motion (ROM) improved or stayed within the normal range in 5 patients within 7 months from the initial measurements. One dog had decreased stifle extension within 7 months (Table 3). The other 2 patients did not have objective ROM measurements during initial evaluation or follow-up. One dog in the treatment group maintained improved stifle extension 18 months after the initial measurement (Table 3).

**Table 3:** Summary of treatment outcomes after extracorporeal shockwave therapy and rehabilitation therapy in dogs with fibrotic myopathy.

Patient outcome	1			2			3			4			Time (mo)	L	R	Time (mo)	L	R	Time (mo)	L	R	Time (mo)	L	R
Passive stifle extension (degrees)	0 7			160 135	160 135	0 8			143 147	119 120	0 7 10 12 18			119 162 155 157 157	111 135 144 147 150	5 34 46 79 81			166 165 160 153 153	165 165 160 150 148
Thigh girth (cm)	0 4.5			50.9 58	50.2 57.5	0 8			55 55.2		–			0 21 46 79 81			53 53 53.2 55.6	
Visual lameness evaluation (grade I-IV)	5			Less kyphotic		0 18			II I		No change			–		
**Time from initial diagnosis to retirement (mo)**	9			27			41			82		

Patient outcome	5			6			7	8			9	10	Time (mo)	L	R	Time (mo)	L	R		Time (mo)	L	R		
Passive stifle extension (degrees)	0 7 9			164 163 142	163 162 156	0.5 Pre-ECSWT Post-ECSWT				123 147	–	0 311			123 155 150	128 150 155	–	–
Thigh girth (cm)	0 7 19			42.6 43 42.3		–			–	–			–	–
Visual lameness evaluation (grade I-IV)	–			No change			No change	3 5			Pathognomonic gait less pronounced No change		–	–
**Time from initial diagnosis to retirement (mo)**	24			6			36	*13			24	1

*Still working full-time at the time of publication. ↑, Improved; ↓, Regressed; –, Not Available; L, Left; R, Right; mo, months.

Three out of the 4 patients who were measured had improved or maintained thigh girth within 4.5 months from the initial measurements. Out of those 3, one had regressed slightly at the 19-month recheck. The other patient initially declined in the thigh girth, and then improved (Table 3).

Three dogs had improved subjective lameness evaluation (less kyphotic stance, or decreased lameness grade from II/IV to I/IV, or less pronounced pathognomonic gait). The other 5 dogs did not have specified gait/lameness change (Table 3).

On average, dogs who received ECSWT and RT were able to work full-time for an additional 32.1 months after the diagnosis of fibrotic myopathy (range 6–82; SD 23.6) (Table 3). Dog #8 was not included in this calculation because he is still actively working at full capacity (13 months since time of diagnosis).

On average, dogs who did not receive ECSWT or RT were able to work full-time for an additional 12.5 months (range 1–24; SD 11.5).

One of the 2 dogs in the non-treatment group was able to work full-time for 24 months with limitations (could not jump in and out of a car or climb stairs). The other dog retired soon after the diagnosis (within 1 month) because he was not able to jump into the patrol vehicle and this disqualified him from being able to work.

No activity limitation was reported for patients who received ECSWT and RT, except that one handler limited jumping due to concern for making the contralateral leg worse. Working duties of the dogs included explosive detection, patrol, and guiding for the blind.

The follow-up for this retrospective study was completed by either phone call or email, 9 months to 7 years after the last treatment. For the non-treatment group, follow-up for one was 2.5 years and the other 10 months after the last evaluation.

Study patients had other comorbidities listed in the medical record including intervertebral disk disease, osteoarthritis, hip pain, iliopsoas pain, tail pain, and hemangiosarcoma. Since we could not obtain the official deposition record, we could not confirm the exact reason for each dog’s retirement.

## Discussion

The results of this retrospective study on 10 working dogs with hindlimb fibrotic myopathy suggested that the combination of ECSWT and RT may allow dogs to continue their working capacity for an average of 32.1 mo (range 6–82; SD 23.6) from the time of diagnosis, thereby confirming our hypothesis. We were not able to statistically compare outcomes with the non-treatment group due to low sample size (2 patients); however, dogs receiving ECSWT and RT in this retrospective series were able to work on average 19.6 mo longer (SD 26.3) as compared to the non-treatment group. While several other modalities were performed, ECSWT was the only consistent modality that all dogs received, in addition to therapeutic exercises and manual therapy (Table 2). This report is the first to describe non-invasive medical treatments that may extend the working ability of dogs diagnosed with fibrotic myopathy of gracilis, semimembranosus, and/or semitendinosus.

Theories regarding the mechanisms of ECSWT to aid in healing of fibrotic myopathy are variable. In terms of human plantar fibromatosis, it is thought that ECSWT stimulates biosynthesis of the extracellular matrix by tendon fibroblasts, which could help in counteracting the maturation process of myofibroblasts and lead to reduced tissue contraction (18). Other theories include that ECSWT causes direct damage to the lesion triggering a healing response, and ECSWT increases vascularity to the lesion, lysing the lesion and resulting in macrophage removal (20). Studies have also demonstrated that ECSWT may inhibit transforming growth factor-β1 (TGF-β1), which plays an important role in enhancing muscle fibrosis (21–24).

Pain reduction by the ECSWT has also been documented in people with fibromatosis diseases (15, 17, 18). Even though fibrotic myopathy usually does not elicit lameness secondary to pain, it can cause pain when the muscle/tendon is stretched above the physiological range. While it was not reported in the patients of this study, in one report (4), most dogs showed pain responses with digital pressure exerted on the affected muscle(s), abduction of the coxofemoral joint(s), or both.

In addition to ECSWT, manual therapy, including massage, passive joint range of motion, and stretching can help collagen/scar tissue to align properly and decrease pain, and is important to help increase flexibility and improve muscle extensibility (25). Therapeutic exercises, focusing on active joint range of motion, warm-up, stretching, and muscle strengthening can also help increase flexibility and prevent future muscle strain. The outcome of the patients presented here likely benefited from these rehabilitation strategies.

Historically, clients/handlers have been told that working dogs’ careers are over when they are diagnosed with fibrotic myopathy. Through client education, they understand that their dogs can continue to work without pain and/or causing additional harm. Additionally, the instruction for activity modification and home exercise program might delay worsening of the contracture and improve functional mobility through required tasks.

Objective gait evaluations using the pressure-sensitive walkway system were only conducted in patients #3 and #4. Inconsistent variations of results were recorded, likely due to the nature of mechanical lameness with fibrotic myopathy. Therefore, this data was not included. Other treatment outcomes, including muscle texture, stifle extension, thigh girth, and subjective lameness exam, did not yield consistent positive results. Those measurements did not correlate with extending the working lifespan of each dog either.

In this retrospective study, we did not assign the severity of the fibrotic myopathy as there is currently no published grading system available, either in clinical examination, or diagnostic imaging modalities (MRI, MSK US), or histopathological evaluation. Despite inconsistent phenotypic improvement, future studies might evaluate the change in fiber pattern with serial MRI or MSK US of the affected muscles to elucidate the effects of ECSWT and RT on canine fibrotic myopathy.

Human studies have used MSK US to evaluate the outcomes of musculoskeletal conditions following ECSWT (18, 26). In one study regarding plantar fibromatosis, the researchers did not find significant changes in length and width of fibroma with sonogram. Reduction in the thickness of the lesion and long-term benefit in pain relief and functional outcomes were noted though (18). In future veterinary studies, the addition of MSK US to follow changes in contracture fibrosis size or progression is recommended.

Working dogs, including military working dogs (MWDs), other federally owned working dogs, police working dogs, and service/guide dogs, provide crucial functions in national defense, public safety, and personal assistance. Maximizing longevity of their service is not just important for the handlers/owners, but also critical for the financial viability of the institutions. The cost of dog acquisition/training before entering active service ranges from $15,454 to $85,000 (27–29). In a study by Moore et al. (30), mean age at death for MWDs in active service was 10 years. The working life of the guide dog is estimated to be 8 years (28). Since fibrotic myopathy is a disease of young adult dogs, increasing the workability of working dogs by an average of 32.1 months with the ECSWT and RT is a significant benefit for the institutions and the handlers.

Limitations of this study are primarily related to the retrospective nature of this case series report including incomplete and inconsistent objective evaluations, and inconsistent rehabilitation treatments that make meaningful comparisons challenging. Recall bias from the owners/handlers could also influence the conclusion because the time and causes of retirement could not be independently verified without the official disposition records, as they are proprietary information. Another limitation is the small number of patients that may not represent the true demographics of the working dog population (male dogs may simply be over-represented in this study), as well as outcomes that may not be repeatable or be different at another rehabilitation facility. A prospective study should be considered in the future, especially evaluation of ECSWT as a potential definitive treatment option for fibrotic myopathy.

Additionally, the absence of an appropriate control group (both in terms of patient numbers and objective outcome measures) is another significant limitation. The low incidence of the disease (or lack of recognition) and lack of standards on assessing workability in working dogs, contributes to the inability of designating an appropriate control group that may conclusively support the use of ECSWT and RT.

While our outcome measures did not demonstrate consistent improvement among the patients, to the authors’ knowledge, there is no standardized functional assessment for police or MWDs with various jobs, including explosive detection and patrol. In particular, we have no objective outcome measures that can assess or predict when a working dog can return to full function and work duties following injury. Recent work by Farr et al. at the Penn Vet Working Dog Center in 2020, provides assessment of a working dog’s foundational fitness (31). Additional studies and refinement will help to provide better assessment as to whether a working dog can continue to work in different functions, given they have different demands in different jobs. Currently, the return-to-work assessment is determined by the veterinarian, handler, and immediate supervisor (or kennel master).

In conclusion, this retrospective case series may support the use of ECSWT and RT for maintaining working capacity of dogs after diagnosis of hindlimb fibrotic myopathy; however, further studies are needed before definitive treatment recommendations can be made.

## Data availability statement

The original contributions presented in the study are included in the article/supplementary material, further inquiries can be directed to the corresponding author.

## Ethics statement

Ethical approval was not required for the study involving humans in accordance with the local legislation and institutional requirements. Written informed consent to participate in this study was not required from the participants or the participants’ legal guardians/next of kin in accordance with the national legislation and the institutional requirements. Ethical approval was not required for the studies involving animals in accordance with the local legislation and institutional requirements because of the retrospective nature of the study: handlers have consented to have their working dogs included in the study. Written informed consent was obtained from the owners for the participation of their animals in this study.

## Author contributions

FT: Data Curation, Writing – original draft, Writing – review & editing. LA: Conceptualization, Formal Analysis, Writing – review & editing.
